# Simultaneous Optimization of Carrier Concentration and Alloy Scattering for Ultrahigh Performance GeTe Thermoelectrics

**DOI:** 10.1002/advs.201700341

**Published:** 2017-09-30

**Authors:** Juan Li, Zhiwei Chen, Xinyue Zhang, Hulei Yu, Zihua Wu, Huaqing Xie, Yue Chen, Yanzhong Pei

**Affiliations:** ^1^ Interdisciplinary Materials Research Center School of Materials Science and Engineering Tongji University 4800 Caoan Road Shanghai 201804 China; ^2^ Department of Mechanical Engineering The University of Hong Kong Pokfulam Road Hong Kong SAR 999077 China; ^3^ School of Environmental and Materials Engineering Shanghai Second Polytechnic University 2360 Jinhai Road Shanghai 201209 China

**Keywords:** alloy scattering, carrier concentration, GeTe, lattice thermal conductivity, thermoelectric

## Abstract

In order to locate the optimal carrier concentrations for peaking the thermoelectric performance in p‐type group IV monotellurides, existing efforts focus on aliovalent doping, either to increase (in PbTe) or to decrease (in SnTe and GeTe) the hole concentration. The limited solubility of aliovalent dopants usually introduces insufficient phonon scattering for thermoelectric performance maximization. With a decrease in the size of cation, the concentration of holes, induced by cation vacancies in intrinsic compounds, increases rapidly from ≈10^18^ cm^−3^ in PbTe to ≈10^20^ cm^−3^ in SnTe and then to ≈10^21^ cm^−3^ in GeTe. This motivates a strategy here for reducing the carrier concentration in GeTe, by increasing the mean size of cations and vice‐versa decreasing the average size of anions through isovalent substitutions for increased formation energy of cation vacancy. A combination of the simultaneously resulting strong phonon scattering due to the high solubility of isovalent impurities, an ultrahigh thermoelectric figure of merit, *zT* of 2.2 is achieved in GeTe–PbSe alloys. This corresponds to a 300% enhancement in average *zT* as compared to pristine GeTe. This work not only demonstrates GeTe as a promising thermoelectric material but also paves the way for enhancing the thermoelectric performance in similar materials.

## 1 Introduction

Due to the increasing energy demand, thermoelectric technology, which enables a direct conversion between heat and electricity,^1^ has drawn increasing attentions in recent decades. The energy conversion efficiency depends on materials' thermoelectric dimensionless figure of merit, *zT* = (*S* ^2^ *T*)/(ρ(κ_E_ + κ_L_)). Here, *S*, *T*, ρ, κ_E_, κ_L_ are Seebeck coefficient, absolute temperature, electrical resistivity, electronic, and lattice contribution to thermal conductivity, respectively.

In order to enhance *zT*, successful electronic approach is typified by band structure engineering, including band convergence,^2^ low band effective mass,^3^ and a weak scattering^4^ are applied to decouple the strongly correlated Seebeck coefficient, resistivity and electronic thermal conductivity. While effective thermal approach focuses on microstructure engineering for phonon scattering sources such as nanostructuring,^5^ dislocations,^6^ lattice anharmonicity,^7^ liquid phonons,^8^ vacancy,^9^ substitutional^10^ or interstitial point defects,^11^ low sound velocity^12^ for minimizing the lattice thermal conductivity.

All the above strategies guarantee the highest possible *zT* only if the carrier concentration is fully optimized. This is because, for all thermoelectrics, both the power factor (*S* ^2^/ρ) and *zT* can be maximized only in a narrow energy range of Fermi level,^13^ which corresponds to a narrow range of carrier concentration. It is further known that the required carrier concentration maximizing the thermoelectric performance is strongly temperature and density of states effective mass dependent.[[qv: 13a,14]] Control of the carrier concentration, is usually enabled by chemical doping, therefore, optimization of thermoelectrics fundamentally requires a detailed assessment on the capability of doping.

Optimal carrier concentration usually locates in the range of 10^19^–10^21^ cm^–3^ for most of known thermoelectrics.^15^ This requires a similar concentration of dopant, since each dopant atom usually releases one electron or hole in most of doped thermoelectrics. These dopants should introduce additional phonon scattering due to the mass and size fluctuations between the dopant and host atoms, but the resulting impurity concentration is usually insufficient for a simultaneous minimization in lattice thermal conductivity. Therefore, well improved thermoelectrics usually involve, not only an optimization of carrier concentration,[[qv: 15a–c]] but also additional strategies enabling a further reduced lattice thermal conductivity.^10^, ^12^, ^16^

With a decrease in the size of cation, the concentration of cation vacancies in intrinsic PbTe, SnTe, and GeTe thermoelectric compounds with a large sized anion framework, increases by orders of magnitude.^17^ This leads to the intrinsic holes increases rapidly from ≈10^18^ cm^−3^ in PbTe to ≈10^20^ cm^−3^ in SnTe and then to ≈10^21^ cm^−3^ in GeTe. Existing efforts, either to increase (in PbTe) or to decrease (in SnTe and GeTe) the carrier concentration, focus on chemical doping by aliovalent impurities for thermoelectric applications.

The size of cation in the compounds mentioned above, seems to be closely related to the intrinsic carrier concentration. This implies a strategy for reducing (means optimization here) the carrier concentration in GeTe, by increasing the mean size of cations and vice‐versa decreasing the average size of anions through isovalent substitutions. Such an effect can be seen in SnTe, where an isovalent substitution of Sn by smaller Mn,^18^ Mg,^19^ and Ga^20^ increases the carrier concentration while a bigger cation substitution by Cd^21^ decreases. Similarly in PbTe, cation substitution by a smaller sized Sn leads to an increase in carrier concentration.^22^ It should be noted that, the change in carrier concentration by isovalent substitutions is unnecessarily as significant as that by aliovalent doping. This enables both an optimized carrier concentration and a well‐reduced lattice thermal conductivity to be achieved in the same concentration of isovalent substitutions. The difference on the cation size might further relate to the phase transitions in SnTe and GeTe. In more details, with a decreased cation size from PbTe to SnTe and then to GeTe, a NaCl structure can only be stabilized in SnTe and GeTe at temperatures above 100^23^ and 720 K,^24^ respectively.

With a carrier concentration optimization only, GeTe has recently been found to show a *zT* as high as 1.7, because of the favorable band structures for thermoelectrics in both low and high temperature phases.^25^ In order to ensure a sufficient phonon scattering by point defects in this material, high concentration impurities, with large differences in both size and mass, as compared to those of the host atom are desired. This makes Pb ideal for substituting Ge, and indeed leads to an observation of a high thermoelectric performance in GeTe–PbTe alloys.^26^ However, the solubility of PbTe is limited to be only ≈13%,^27^ presumably due to the large lattice mismatch between GeTe and PbTe. This leaves possibilities for further improvements by a further reduction in the lattice thermal conductivity through alloying.

Since the lattice mismatch between GeTe and PbSe is smaller as compared to that of GeTe versus PbTe. This might be favorable for a higher solubility of PbSe in GeTe. It is observed a higher concentration of Pb substitution can be achieved when half of Te is substituted by Se.^28^ Therefore, alloying GeTe with PbSe is very likely to increase the concentration of Pb. In addition, Se substitution on Te site would also create point defects. Both cation and anion substitutional defects should introduce strong phonon scattering for a minimal lattice thermal conductivity. Further due to the size effect, the increase in the average size of cations and the decrease in the mean size of anions in GeTe–PbSe alloys, are expected to be favorable for reducing the concentration of cation vacancies, and therefore the carrier concentration to approach its optimum.

In this work, efforts are devoted to optimizing the carrier concentration and reducing the lattice thermal conductivity of GeTe by alloying with PbSe. Such a simple alloying process, nicely realizes a continuous control of carrier concentration crossing its optimum. Being different from existing chemical doping by aliovalent dopants, alloying with PbSe ensures an optimal carrier concentration at a much higher concentration of substitutional defects. This leads to, an extremely low lattice thermal conductivity of ≈0.5 W (m K)^−1^ in the entire temperature range, and eventually an ultrahigh thermoelectric figure of merit, *zT* of 2.2 in simple solid solutions. This work enables new possibilities for optimizing thermoelectric performance in GeTe and similar materials.

## 2 Results and Discussion

**Figure 1:**
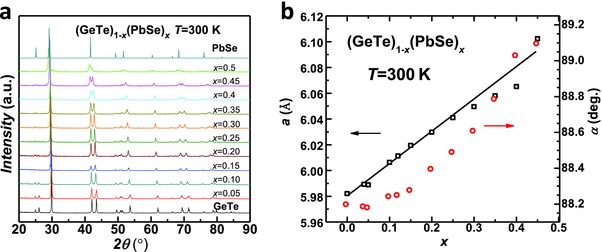
Room temperature a) X‐ray diffraction patterns and b) lattice parameters and interaxial angle for (GeTe)_1−_ *_x_*(PbSe)*_x_* solid solutions.

The room temperature XRD patterns for (GeTe)_1–_ *_x_*(PbSe)*_x_* are shown in **Figure** **1**a. It can be seen that when *x* ≤ 0.45 samples crystallize in the low temperature rhombohedral structure of GeTe. When *x* = 0.5, impurity peaks can be observed, therefore the solubility of PbSe in rhombohedral GeTe is believed to be within 45–50%. Since the rhombohedral structure of GeTe (*a* = *b* = *c* and α = β = γ < 90°) is essentially a slightly distorted rock‐salt lattice along the (111) direction,^29^ the influence on the crystal structure due to alloying can be characterized by the lattice parameters and the interaxial angles, both of which are shown in Figure 1b. The increase in the interaxial angle due to alloying, can be understood by the fact that PbSe crystallizes in a cubic structure.

**Figure 2:**
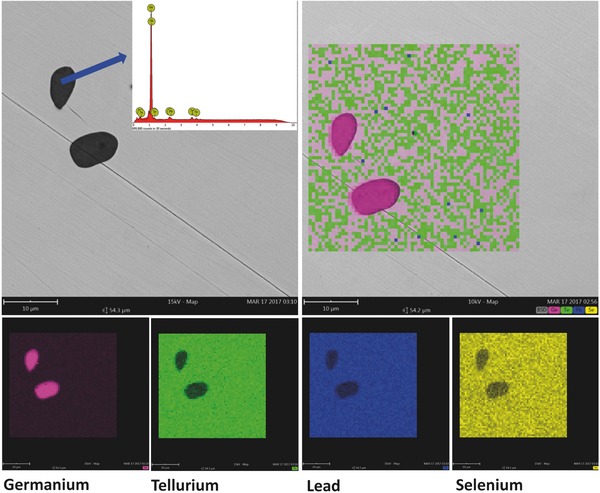
SEM images indicating the existence of Ge precipitates and a uniform distribution of constituent elements in the matrix phase in (GeTe)_0.85_(PbSe)_0.15_.

**Figure 3:**
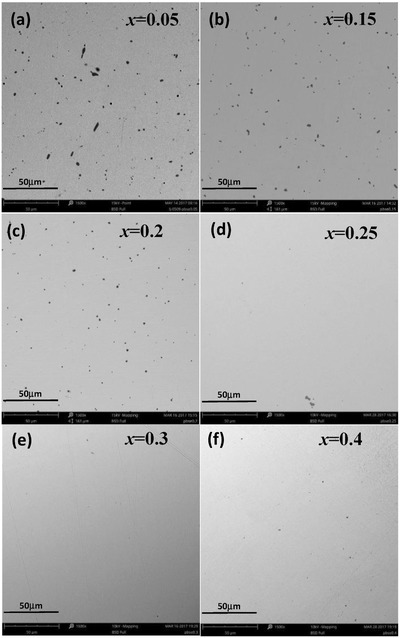
SEM images for (GeTe)_1−_ *_x_*(PbSe)*_x_* showing a removal of Ge precipitates (black) due to PbSe‐alloying.

The lattice parameter increases linearly with increasing *x*, indicating the formation of solid solution, which is confirmed by the SEM observations as shown in **Figures** **2** and **3**. No PbSe precipitates or Pb‐rich phases are observed, and the spacial distributions of both Pb and Se are uniform in the matrix. However, it is seen that high concentration Ge precipitates are observed in the samples with *x* ≤ 20%, which is common in GeTe based materials.[[qv: 26a,28b,30]] It is interesting to note that the concentration of Ge precipitates gradually decreases with increasing *x* and eventually disappears when *x* > 25% (Figure 3).

**Figure 4:**
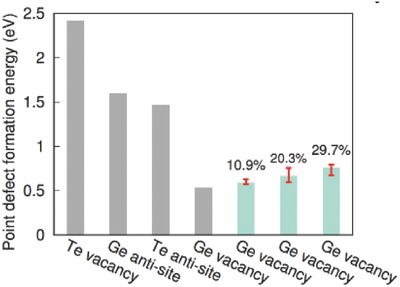
Formation energies of different types of point defects in GeTe (in grey) and that of Ge vacancy in (GeTe)_1−_ *_x_*(PbSe)*_x_* alloys with *x* equals to 10.9%, 20.3%, or 29.7% (in light green). The deviations due to possible occupancies are represented by error bars.

The decrease in the concentration of Ge precipitates, implies a reduced concentration of Ge‐vacancies, indicating increased formation energy of Ge vacancies as confirmed by the DFT calculations. As shown in **Figure** **4**, Ge vacancy shows the lowest formation energy among all possible types of point defects in GeTe, indicating itself as the dominant point defects in this compound. Alloying with PbSe, leads to an increase in the formation energy of Ge vacancies with increasing alloying level. More details about the calculation can be found in the Supporting Information. Experimentally, alloying with PbSe increases the size of cation and decreases the size of anion, both effects tend to reduce the carrier concentration, meaning a reduced concentration of Ge vacancy and thus an increase in Ge solubility.

**Figure 5:**
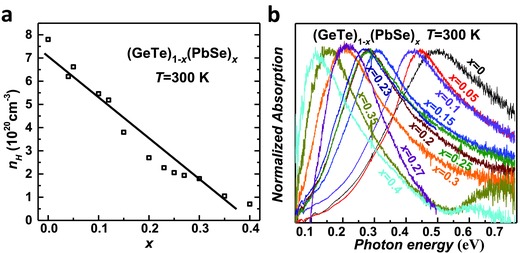
a) Hall carrier concentration versus alloying concentration and b) the normalized absorption versus photon energy for (GeTe)_1−_ *_x_*(PbSe)*_x_* at room temperature.

The increased formation energy of Ge vacancy, and therefore a reduced concentration, is expected to reduce the hole concentration, which is confirmed by the Hall measurement as shown in **Figure** **5**a. The Hall carrier concentration decreases linearly with increasing *x*. This enables a precise control of carrier concentration in a broad range for the GeTe. Importantly, the composition ensuring an optimal carrier concentration of ≈2 × 10^20^ cm^−3^ for GeTe^25^ achieves an extremely high concentration of 30% (≈10^22^ cm^−3^) substitutional defects on both cation and anion sites, which are quite important to guarantee a well‐reduced lattice thermal conductivity simultaneously.

The gradually decreased carrier concentration leads to a shift of the optical absorption by charge carriers to lower energies in (GeTe)_1−_ *_x_*(PbSe)*_x_* solid solutions, as shown in Figure 5b. The normalized absorption shows a maximum at lower energies with increasing *x*, indicating a lowered Fermi level. The optical measure further enables an estimation of inertial effective mass (*m*_I_*), according to the Lyden method^31^ via $\omega_{p}=\sqrt{\frac{ne^{2}}{\varepsilon_{\infty }\varepsilon_{0}m_{I}^{*}}}$. Here, ω_p_ is the angular frequency of the absorption maximum, *n* is the carrier concentration, *e* is the electronic charge, ε_∞_ = 30^32^ is the relative dielectric constant at the high frequency limit, ε_0_ is the permittivity of free space. The resultant inertial effective masses for different carrier concentrations are listed in Table S1 in the Supporting Information, together with literature data for comparison.^32^ This simple approximation yields an average *m*_I_* of ≈0.24 *m*_e_, which shows excellent agreement with that by ab initio calculation (0.25 *m*_e_).^25^

**Figure 6:**
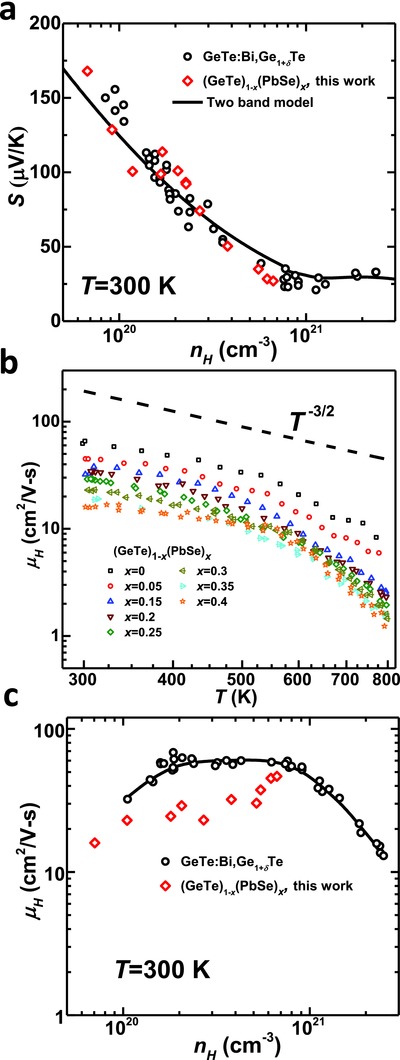
a) Room temperature carrier concentration dependent Seebeck coefficient, b) temperature dependent hall mobility, and c) room temperature carrier concentration dependent Hall mobility for (GeTe)_1−_ *_x_*(PbSe)*_x_*, with a comparison to those of Bi‐doped GeTe.^25^ A dominant carrier scattering by acoustic phonons is shown by the dashed curve in (b) and the black curve in (a) shows a two‐band model prediction.^25^

The room temperature Hall carrier concentration dependent Seebeck coefficient (**Figure** **6**a) for GeTe–PbSe solid solutions, shows no obvious deviations from that of Bi‐doped GeTe and agrees well with the two band model prediction.^25^ This indicates a negligible modification of the band structure due to PbSe‐alloying. The model prediction, based on an acoustic scattering as evidenced from the temperature dependent Hall mobility measurements (Figure 6b). The carrier concentrations obtained here ranges from 7 × 10^19^ to 8 × 10^20^ cm^−3^, which locates effectively in the single band region according to Figure 6a. Due to the existence of high concentration point defects, solid solutions show a slightly reduced Hall mobility (Figure 6c), which is normally seen in many thermoelectric alloys including PbTe,[[qv: 2c,g,33]] SnTe,^34^ and PbSe.^4^

**Figure 7:**
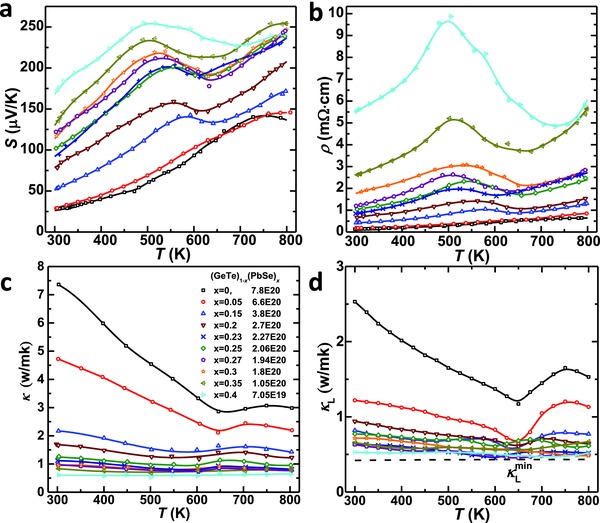
Temperature dependent a) Seebeck coefficient, b) resistivity, c) thermal conductivity, and d) lattice thermal conductivity for (PbSe)*_x_*(GeTe)_1−_ *_x_*.

Temperature dependent Seebeck coefficient, resistivity, thermal conductivity, and its lattice component are shown in **Figure** **7**. It is seen that majority of the samples studied here show a degenerated semiconducting behavior, meaning an increase in both resistivity and Seebeck coefficient with increasing temperature. Due to the decrease in carrier concentration by PbSe‐alloying, both Seebeck coefficient and resistivity increase with increasing *x*. The sudden changes in Seebeck coefficient and resistivity are observed in the temperature range of 450–650 K, depending on the composition. This is due to the phase transition induced switch in energy position between *L* and *Σ* valence bands.^25^

The lattice thermal conductivity (κ_L_) is estimated by subtracting the electronic component (κ_E_ = *LT*/ρ) from the total thermal conductivity, where the Lorenz factor (*L*) is estimated based on a single Kane band model approximation. Due to the strengthened phonon scattering by point defects, the lattice thermal conductivity decreases with increasing *x* and shows a maximal reduction of 75%. The lowest κ_L_ achieved in this work is about 0.5 W (m K)^−1^, which approaches the amorphous limit of GeTe according to the Cahill model.^35^ For the sample (GeTe)_0.6_(PbSe)_0.4_, the concentration of substitutional point defects is high enough to ensure a lattice thermal conductivity approaching the amorphous limit of GeTe at all measured temperatures, leading the κ_L_ to be nearly temperature independent.

**Figure 8:**
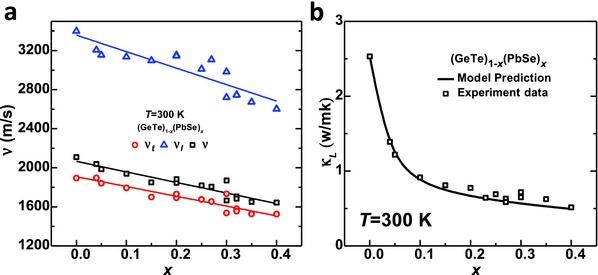
a) Composition dependent sound velocity and b) lattice thermal conductivity for (GeTe)_1−_ *_x_*(PbSe)*_x_* at 300 K. A model prediction (black curve) on lattice thermal conductivity agrees well the experiment results.

To quantitatively understand the influences of Pb/Ge and Se/Te substitutions on the lattice thermal conductivity, room temperature sound velocity of the samples are measured (**Figure** **8**a). It is seen that both transverse (*v*_t_) and longitudinal (*v*_l_) sound velocities decrease linearly with increasing PbSe content. Taking into account the change in sound velocities due to alloying, a Debye model^36^ is developed to predict the composition dependent lattice thermal conductivity. The model considers phonon–phonon and point defect scattering, with more details given in the Supporting Information. It is seen that the experimentally observed reduction in the lattice thermal conductivity can be well predicted by this model (black curve in Figure 8b), indicating the introduced point defects in GeTe–PbSe solid solutions are indeed responsible. It should be noted that PbSe‐alloying does not induce a decrease in band gap, and therefore, none of the samples here shows a significant influence on transport properties due to bipolar effect.

**Figure 9:**
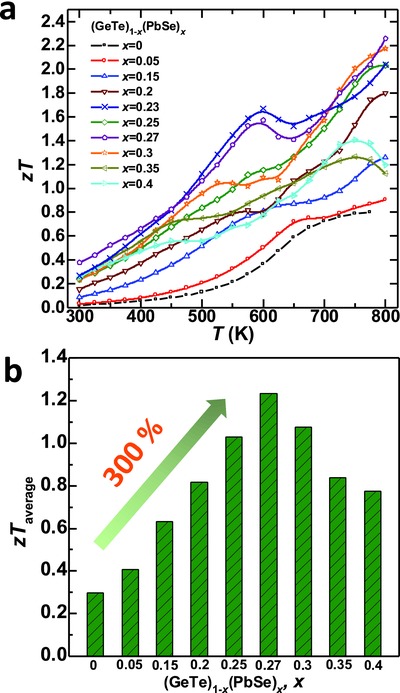
Temperature dependent thermoelectric figure of merit, a) *zT* and b) their average for (GeTe)_1−_ *_x_*(PbSe)*_x_*.

**Figure 10:**
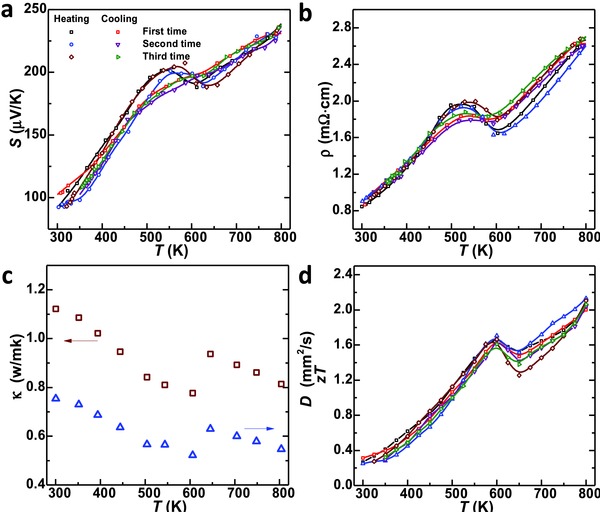
Repeated measurements on temperature dependent a) Seebeck coefficient, b) resistivity, c)thermal conductivity and diffusivity, and d) thermoelectric figure of merit for the high *zT* composition (GeTe)_0.73_(PbSe)_0.27_, indicating a highly reproducible performance.

Due to the dual effects of both optimizing the carrier concentration and reducing the lattice thermal conductivity, the thermoelectric figure of merit, *zT*, is found to be up to 2.2 (**Figure** **9**a). This further leads to an average *zT* > 1.2 (Figure 9b) for (PbSe)_0.27_(GeTe)_0.73_. With an increase in PbSe concentration, the average *zT* climbs up, and reaches its maximum when *x* = 0.27. When *x* is within 0.23–0.30, the Hall carrier concentration is about ≈2 × 10^20^ cm^−3^, approaching its optimum for GeTe.^25^, ^37^ Therefore a peak *zT* > 2.0 can be guaranteed at 800 K. Comparing to the pristine GeTe, alloying with 27% PbSe enables a 300% enhancement in the average *zT*, strongly indicating the effectiveness of alloying for performance improvements in thermoelectric GeTe. Moreover, the high *zT* material is shown to be highly stable, as confirmed by the reproducible properties measured by several times during both heating and cooling thermal cycles in a resynthesized (GeTe)_0.73_(PbSe)_0.27_ (**Figure** **10**).

## 3 Summary

In summary, alloying GeTe with PbSe enables isovalent substitutions at both cation and anion sites, for a precise control on the carrier concentration, through the control of intrinsic cation vacancies. The fully optimized carrier concentration is achievable, in a composition with sufficient substitutional defects for an effective reduction in the lattice thermal conductivity simultaneously. The dual effects of both tuning the carrier concentration and scattering the phonons, eventually enhance the thermoelectric figure of merit, *zT*, up to 2.2 in alloy materials, which corresponds to an improvement by 300% in the average *zT* as compared to pristine GeTe. The obtained lattice thermal conductivity approaches the amorphous limit, due to the high concentration substitutional defects and the large size and mass difference between the host and impurity atoms. This work demonstrates that isovalent substitution is a simple yet effective approach for thermoelectric performance improvements in GeTe and similar materials.

## 4 Experimental Section

Polycrystalline (GeTe)_1–_ *_x_*(PbSe)*_x_* were synthesized with high purity elements (>99.99%). The raw materials were sealed in vacuum quartz ampoules and melted at 1223 K for 6 h. Then a cold water quenching and an annealing at 873 K for 3 d were carried out. The annealed ingots were hand‐ground into fine powders (a few micrometer in diameter) for densification at 853 K for 40 min under a uniaxial pressure of ≈80 MPa, using an induction heating hot press.^38^ X‐ray diffraction (XRD, DX2700) and infrared diffuse reflection spectrum (Bruker Tensor II equipped with Diffuse Reflectance attachment) were carried out for powders at room temperature. Optical reflectance was measured by Fourier Transform Infrared Spectroscopy (FTIR, Bruker Tensor II equipped with a Diffuse Reflectance attachment) at room temperature. Microstructure was characterized by scanning electron microscope (SEM, Phenom Pro) equipped with energy dispersive spectroscopy. Then the powders were hot pressed into pellets with ≈12 mm in diameter and ≈1.5 mm in thickness and a density of >≈98% for transport property measurements.

The thermal diffusivity (*D*) was measured by a Netzsch LFA457 laser flash system. A Dulong–Petit limit of heat capacity (*C*_p_) is used to determine the thermal conductivity via κ = *dC*_p_ *D*, where *d* is the density estimated by mass/volume of the pellets. Resistivity, Seebeck and Hall coefficients were simultaneously measured on the pellets, where the hot and cold side temperatures were measured by two K‐type thermocouples attached to the opposite edges of the pellet sample and the thermopower was measured by two Nb wires welded to the thermocouple tips. A four‐probe Van der Pauw technique was used for both resistivity and Hall effect measurement. The maximal magnetic field was 1.5 T with both positive and negative polarizations. The slope of the thermopower versus temperature gradient 0–5 K^39^ enabled an estimation of the Seebeck coefficient. All the measurements of transport properties were carried out from 300 to 800 K, and the reproducibility was confirmed by multiple measurements on the same sample and in multiple samples with similar carrier concentrations. Longitudinal and transverse sound velocities were determined by pulse‐receiver (Olympus‐NDT) equipped with an oscilloscope (Keysight).

First‐principles calculations based on density functional theory (DFT) were performed using the Vienna ab initio simulation package^40^ for characterizing the formation energies of different types of point defects in GeTe and GeTe–PbSe alloys. The Perdew–Burke–Ernzerhof functional of the generalized gradient approximation was applied within the projector‐augmented wave method.^41^ An energy cut‐off of 300 eV was used in all DFT calculations. Brillouin zone was sampled with a density of about 2π × 0.02 Å^−1^ adopting the Γ‐centered Monkhorst–Pack scheme. The electronic convergence criterion was set to 10^−6^ eV. Generalized quasirandom structures containing 128 atoms of GeTe alloyed with different concentrations of PbSe were obtained by minimizing the structural order using the evolutionary algorithm.^42^ One point defect was introduced in the quasirandom supercell to calculate the formation energy, while four generalized quasirandom structures with similar structural orders have been considered for (GeTe)_1−_ *_x_*(PbSe)*_x_* alloys. All the supercells were fully relaxed before self‐consistent total energy calculations.

## Conflict of Interest

The authors declare no conflict of interest.

## Supporting information

Supplementary
