# Supplementary material for: Simultaneous Optimization of Carrier Concentration and Alloy Scattering for Ultrahigh Performance GeTe Thermoelectrics
Source: Adv Sci (Weinh). 2017 Sep 30;4(12):1700341. doi: 10.1002/advs.201700341 (PMC5737105; doi:10.1002/advs.201700341)
Supplement: Supplementary file 1 — Supplementary [file ADVS-4-na-s001.pdf]

Copyright WILEY-VCH Verlag GmbH & Co. KGaA, 69469 Weinheim, Germany, 2017.

ADVANCED SCIENCE
Open Access

Supporting Information

for *Adv. Sci.,* DOI: 10.1002/advs.201700341

Simultaneous Optimization of Carrier Concentration and Alloy Scattering for Ultrahigh Performance GeTe Thermoelectrics

*Juan Li, Zhiwei Chen, Xinyue Zhang, Hulei Yu, Zihua Wu, Huaqing Xie, Yue Chen, and Yanzhong Pei**

Supplementary

Simultaneous optimization of carrier concentration and alloy scattering for ultrahigh performance GeTe thermoelectrics

Juan Li[1], Zhiwei Chen[1], Xinyue Zhang[1], Hulei Yu[2], Zihua Wu[3], Huaqing Xie[3], Yue Chen[2], and Yanzhong Pei[1*]

[1]Interdisciplinary Materials Research Center, School of Materials Science and Engineering, Tongji Univ., 4800 Caoan Rd., Shanghai 201804, China.

[2]Department of Mechanical Engineering, The University of Hong Kong, Pokfulam Road, Hong Kong SAR, China

[3]School of Environmental and Materials Engineering, Shanghai Second Polytechnic Univ., 2360 Jinhai Rd., Shanghai, 201209, China,

[*]Email: yanzhong@tongji.edu.cn

Calculation of the formation energy of point defects:

The point defect formation energies in GeTe were calculated according to[1]:

$$E_f = E\left(\mathrm{Ge}_{64-x}\mathrm{Te}_{64-y}\right) - E\left(\mathrm{Ge}_{64}\mathrm{Te}_{64}\right) + x\mu(\mathrm{Ge}) + y\mu(\mathrm{Te}),$$

where $E\left(\mathrm{Ge}_{64-x}\mathrm{Te}_{64-y}\right)$ and $E\left(\mathrm{Ge}_{64}\mathrm{Te}_{64}\right)$ represent the total energies of a defective and a perfect supercell, respectively. μ denotes the chemical potential of an element in its bulk phase. The formation energies of a Ge vacancy in GeTe-PbSe alloys were calculated using the following equation:

$$E_f^{V_{\mathrm{Ge}}} = E\left(\mathrm{Ge}_{63-x}\mathrm{Pb}_x\mathrm{Te}_{64-x}\mathrm{Se}_x\right) - E\left(\mathrm{Ge}_{64-x}\mathrm{Pb}_x\mathrm{Te}_{64-x}\mathrm{Se}_x\right) + \mu(\mathrm{Ge}).$$

It is seen from Fig. 4 that alloying GeTe with PbSe increases the Ge vacancy formation energy; i.e., Ge vacancy becomes more difficult to form in GeTe-PbSe alloys, which is in agreement with the experimentally observed decrease of hole carrier concentration.

Supplementary Table 1. The composition, Hall carrier concentration, inertial effective mass, plasma frequency, the corresponding energy and the density of samples.

**Composition $(\mathrm{GeTe})_{1-x}(\mathrm{PbSe})_x$**	**Carrier concentration (10^{20}cm^{-3})**	**Inertial Effective Mass (m_e)**	**Plasma frequency (10^{14}Hz)**	**Plasma energy (eV)**	**Density (g/cm^3)**
x=0	7.8	0.22	7.43	0.489	6.21
x=0.05	6. 7	0.24	6.63	0.436	6.39
x=0.1	5.5	0.20	6.25	0.411	6.20
x=0.15	3.8	0.24	4.82	0.317	6.47
x=0.2	2.7	0.21	4.37	0.287	6.53
x=0.23	2.3	0.20	4.14	0.273	6.66
x=0.25	2.1	0.21	3.92	0.258	6.42
x=0.27	2.0	0.25	3.46	0.228	6.34
x=0.3	1.8	0.29	2.73	0.180	6.49
x=0.35	1.1	0.25	2.56	0.169	6.59
x=0.4	0.7	0.30	1.81	0.119	6.3
	3.0	0.21			
GeTe	3.5	0.23	Ref.[1]		
	5.0	0.28			

Supplementary

Debye Model:

The temperature-dependent lattice thermal conductivity can be estimated with the following equation[3]:

$$\kappa_L = \frac{k_B}{2\pi^2 v}\left(\frac{k_B T}{\hbar}\right)^3 \int_0^{\theta_D/T} \tau_{tot}(x)\frac{x^4 e^x}{(e^x-1)^2}dx \qquad (1)$$

Where k_B is the Boltzman constant, v is average sound velocity, $\hbar$ is the reduced Plank constant, θ_D is Debye temperature, τ_{tot} is the total relaxation time and $x = \hbar\omega/\kappa_B T$.

For $(GeTe)_{1-x}(PbSe)_x$ solid solutions, the total relaxation time includes the contributions from phonon-phonon, point defect and dislocation scattering respectively by:

$$\tau_{tot}^{-1} = \tau_U^{-1} + \tau_N^{-1} + \tau_{PD}^{-1} \qquad (2)$$

$$\tau_U^{-1} = \frac{2}{(6\pi^2)^{1/3}}\frac{\kappa_B \bar{V}^{1/3}\gamma^2\omega^2 T}{\bar{M}v^3} \qquad (3)$$

$$\tau_N^{-1} = \frac{2}{(6\pi^2)^{1/3}}\frac{\kappa_B \bar{V}^{1/3}\gamma^2\omega^2 T}{\bar{M}v^3} \qquad (4)$$

$$\tau_{PD}^{-1} = \frac{\bar{V}\omega^4}{4\pi v^3}\sum_i x_i(1-x_i)\left[\left(\frac{M_i-M}{M}\right)^2 + \varepsilon\left(\frac{a_i-a}{a}\right)^2\right] \qquad (5)$$

The anharmonic parameter ε can be estimated[4-6] by $\varepsilon = \frac{2}{9}\left[(4+6.4\gamma)\frac{1+r}{1-r}\right]^2$, Poisson's ratio[7] $r = \frac{v_l^2 - 2v_t^2}{2(v_l^2 - v_t^2)}$

Supplementary Table 2. Parameters used for the Debye model.

Parameters	Description	Values	Ref.
β	Ratio of N- to U-process	0.52	estimated
$\bar{V}$	Average atomic volume of $(GeTe)_{1-x}(PbSe)_x$	$a_i^3 sin\alpha_i\sqrt{1-\frac{2cos^2\alpha_i}{cos\alpha_i+1}}/8$ m^3	This work
$\bar{M}$	Average atomic mass of $(GeTe)_{1-x}(PbSe)_x$	$M_{(GeTe)1-x(PbSe)x}/(2\times 6.023\times 10^{23})$ kg	-
x_i	Impurity concentration in solid solutions	$x_{PbSe} \leq 0.4$	-
v	Average sound speed	2030.2-954.87 x_i	This work
v_L	Longitudinal sound speed	3358.6-1689.7 x_i	This work
v_T	Transverse sound speed	1908.8-954.87 x_i	This work
γ	Gruneisen parameter	2.19	8
M_i	Atomic mass of impurity	M_{Pb}=207.2 g·mol^{-1}, M_{Se}=78.96 g·mol^{-1}	-
M	Atomic mass of matrix	M_{Ge}=72.64 g·mol^{-1}, M_{Te}=127.6 g·mol^{-1}	-
a_i, α_i	Lattice parameter of $(GeTe)_{1-x}(PbSe)_x$	a_i=0.21773x_i+5.98307 Å<br>α_i=88.1896+0.1458x_i+2.8061x_i^2+5.0480x_i^3(deg.)	This work

Supplementary references:

1. Yue Chen, Jia-Xiang Shang and Y. Zhang, *Intermetallics*, 2007, **15**, 1558-1563.
2. J. E. Lewis, *Phys. Stat. Sol. (b)*, 1973, **59**, 367-377.
3. J. Callaway and H. C. Vonbaeyer, *Phys Rev*, 1960, **120**, 1149-1154.
4. B. Abeles, *Phys Rev*, 1963, **131**, 1906-1911.
5. P. G. Klemens, *Proceedings of the Physical Society*, 1955, **A68**, 1113-1128.
6. Y. Gelbstein, O. Ben-Yehuda, Z. Dashevsky and M. P. Dariel, *J. Cryst. Growth*, 2009, **311**, 4289-4292.
7. D. J. Singh, *J. Appl. Phys.*, 2013, **113**, 203101.
8. Yaniv Gelbstein, Joseph Davidow, Ehud Leshem, Oren Pinshow and S. Moisa, *Phys. Status Solidi B*, 2014, **251**, 1431–1437.
